# Mahamana Technique of Pancreaticogastrostomy: A Safe and Reliable Technique for a Difficult Pancreatic Stump

**DOI:** 10.7759/cureus.59576

**Published:** 2024-05-03

**Authors:** Mayank Tripathi, Kumar Vineet, Nishant Kumar, Piyush K Shukla

**Affiliations:** 1 Surgical Oncology, Mahamana Pandit Madan Mohan Malviya Cancer Centre (MPMMCC) & Homi Bhabha Cancer Hospital (HBCH), Varanasi, IND

**Keywords:** • pancreatic tumors, hepato-pancreato-biliary surgery, pancreatic-biliary cancer, pancreatico gastrostomy, whipples operation

## Abstract

Pancreatoduodenectomy is a complex surgical procedure involving three anastomoses. Anastomosis of the pancreatic stump with the gastrointestinal tract is associated with most complications described in the postoperative period. So, there have been multiple attempts to discover safe and sound steps for this particular anastomosis. Pancreaticogastrostomy involves anastomosis between the remaining pancreas and stomach. Since it was first performed, its surgical steps have been modified multiple times, but there is no gold standard method to perform it. In this paper, we describe the surgical steps of pancreaticogastrostomy in difficult pancreatic stumps in eight patients using two transpancreatic sutures, one purse string suture, and the incorporation of transpancreatic sutures in the third layer of the gastrojejunostomy anastomosis. Postoperative outcomes of this series have provided encouraging short-term results.

## Introduction

Pancreatoduodenectomy (PD) involves the removal of the head of the pancreas along with parts of the duodenum for various entities like carcinoma of the head of the pancreas, periampullary carcinomas, chronic pancreatitis, distal cholangiocarcinomas, and various other benign and malignant conditions of the pancreatic head and uncinate process [1]. It involves three anastomoses after the completion of resection. Anastomosis between the remaining part of the pancreas and the gastrointestinal tract has remained a topic of debate since the inception of PD surgery due to various postoperative complications. Worldwide surgeons differ in their approach to the anastomosis. Some prefer pancreaticojejunostomy (PJ) while others are more inclined toward pancreaticogastrostomy (PG). After the landmark paper that established pancreaticogastrostomy as a safe and alternative way of anastomosis, there have been many modifications in the technique over the years [2].

The RECOnstruction after PANCreatoduodenectomy Study (RECOPANC) trial established that the rate of postoperative pancreatic fistula (POPF) is not different between PJ and PG but there have been more instances of postoperative pancreatic hemorrhages (PPH) after PG [3]. In a meta-analysis performed in 2017, there were more PPH in PG but the rate of clinically significant PPH was not statistically significant compared to PJ. It also states that postoperative pancreatic exocrine function was better in PG compared to PJ in the first six months and a negligible difference was observed at 12 months after surgery [4]. So in our endeavor to further improve the technique of pancreaticogastrostomy, we describe a modified technique of PG i.e. the Mahamana technique performed in eight patients with encouraging short-term results.

## Technical report

This series comprises eight patients who underwent open PD for adenocarcinomas over four months with PG as a technique of anastomosis performed by the same surgeon for establishing pancreatic and gastrointestinal continuity in a unit of the department of surgical oncology. Classical PD was performed in seven patients and pylorus-preserving PD (PPPD) was done in one patient. Patient data, including demographics, type of anastomosis, characteristics of the intraoperative pancreas, and postoperative events, were retrieved retrospectively from prospectively maintained electronic medical records (Table 1).

**Table 1:** Patients’ characteristics PTBD: percutaneous transhepatic biliary drainage, POD: postoperative day, CBD: common bile duct, FRS: fistula risk score

	Patient 1	Patient 2	Patient 3	Patient 4	Patient 5	Patient 6	Patient 7	Patient 8
Age (yrs)	56	64	59	60	68	63	61	54
Comorbidity	Nil	Diabetes and Hypertension	Diabetes and Hypertension	Diabetes	Nil	Diabetes and Hypertension	Nil	Nil
Preoperative Biliary drainage	Stent	Stent	PTBD	Nil	Stent	Nil	Nil	Nil
Blood loss (ml)	350	400	400	200	300	350	300	1200
Drain amylase POD 3	80	28	30	64	50	48	96	527
POD of discharge	6	9	8	6	7	8	7	10
Site of malignancy	Periampullary	Periampullary	Head of Pancreas	Periampullary	Distal CBD	Periampullary	Periampullary	Distal CBD
FRS	5	6	5	6	7	5	6	9

All the patients have common characteristics of a soft pancreas with a non-dilated pancreatic duct, with a duct size of less than or equal to 3 mm. All patients have one Jackson Pratt drain placed, which was in close proximity to PG. A proton pump inhibitor was given at a dose of 40 mg/day till postoperative day (POD) 10. The amylase activity of the drain was measured on POD 3 and if any drain was remaining till POD 7, the drain amylase level was measured again.

Institutional ethical committee clearance was taken from the Institutional Ethics Committee (IEC), Mahamana Pandit Madan Mohan Malviya Cancer Centre (MPMMCC) & Homi Bhabha Cancer Hospital (HBCH) for the retrospective analysis of all these eight cases. A waiver of consent was given by the Institutional Ethics Committee in view of the retrospective analysis. 

After performing resection in PD, hemostasis was achieved using bipolar cautery or an adequate-size polypropylene suture.

Steps of the Mahamana technique

**Figure 1:**
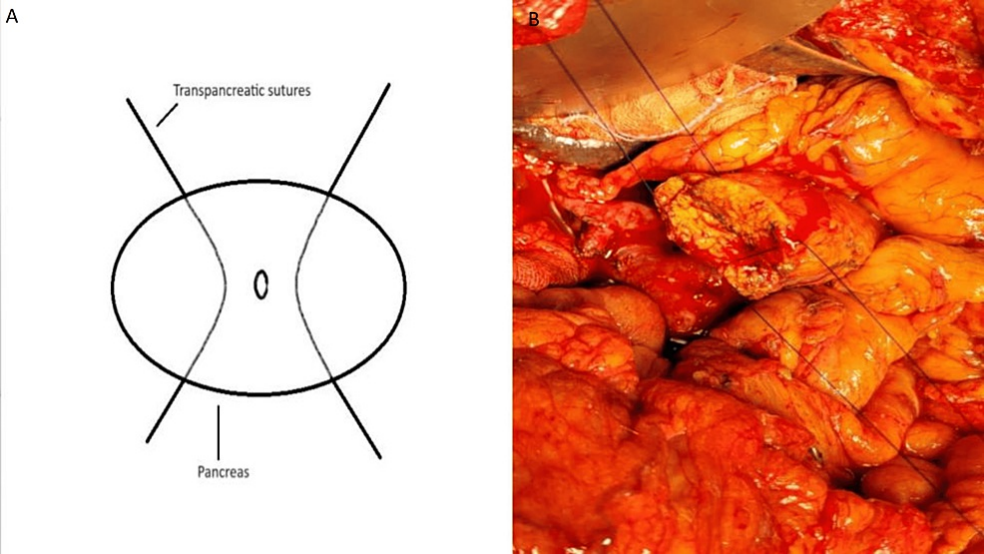
A. A schematic diagram of trans-pancreatic sutures (credit: authors), B. Intraoperative image of trans-pancreatic sutures

**Figure 2:**
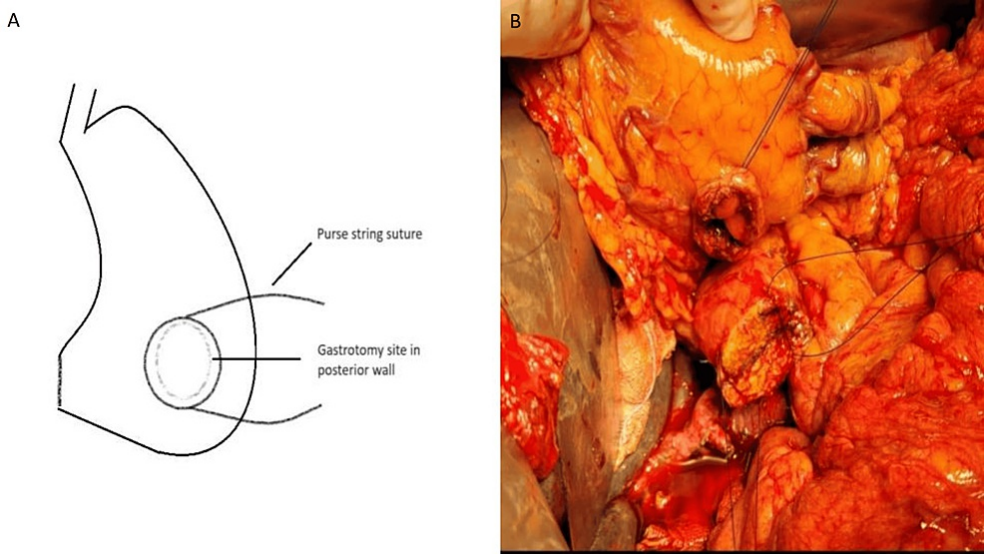
A. A schematic diagram of a purse string suture on the posterior stomach surface (credit: authors), B. Intraoperative image of the purse string suture on the posterior stomach surface

**Figure 3:**
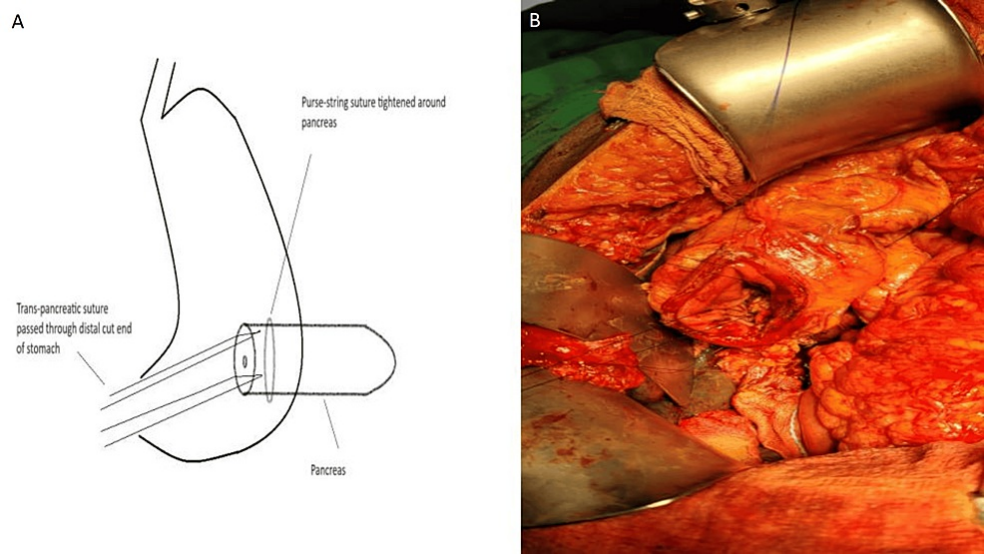
A. A schematic diagram of the passage of the trans-pancreatic suture through the distal cut end of the stomach (credit: authors), B. Intraoperative image of the trans-pancreatic suture passed through the distal cut end of the stomach

**Figure 4:**
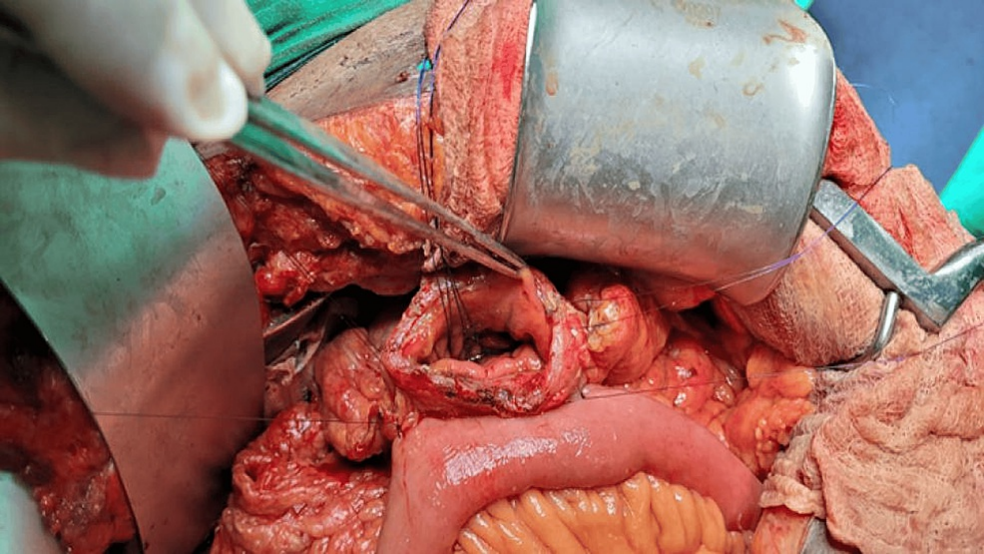
Intraoperative image of the fourth layer of the gastrojejunostomy (GJ)

**Figure 5:**
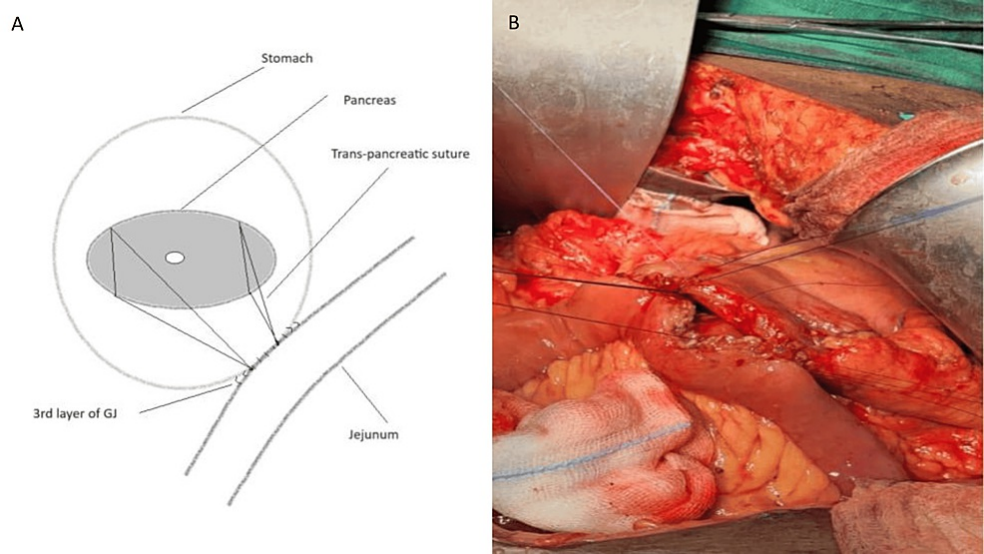
A. A schematic diagram of the incorporation of the trans-pancreatic suture in the third layer of gastrojejunostomy (credit: authors), B. Intraoperative image of incorporation of the trans-pancreatic suture in the third layer of the gastrojejunostomy GJ: gastrojejunostomy

1. The remaining pancreas is mobilized from the surrounding tissue and splenic vessels till adequate mobility is achieved to reach about 3-4 cm.

2. Pancreatic ducts in these cases are very small so identification of ducts is not necessary.

3. Two trans-pancreatic sutures of 3-0 round body polypropylene are taken over the superior and inferior surfaces of the pancreas in a U-like fashion (Figure 1).

4. A suitable-size gastrotomy is made over the posterior surface of the stomach near the cut surface of the pancreas to accommodate the pancreas through it. The length of the gastrotomy needs to be chosen according to the consistency of the pancreas so that it is neither too loose nor too constricting. In most cases, it should not be more than 2 cm.

5. A purse string suture is taken around the gastrotomy using polypropylene 2-0 round body material (Figure 2).

6. The pancreas is mobilized about 3-4 cm. A stapled gastric antrum is opened. A Babcock forceps is passed through this opening and gastrotomy. The pancreatic stump along with two transpancreatic sutures are pulled into the stomach through the gastrotomy. The purse string suture is tightened across the pancreas and stomach wall. The tension of the purse string suture should be adequate to ensure tight adherence of the gastric wall to the remnant pancreatic stump. It should not hamper the blood supply of the remaining pancreas.

7. Both trans-pancreatic sutures are pulled across the distal cut end of the stomach (Figure 3).

8. A Roux-en-Y gastrojejunostomy (GJ) is performed in four layers using a 4-0 round body polydioxanone suture between the jejunum and distal cut end of the stomach (Figure 4).

9. While taking the third layer of the GJ, both trans-pancreatic sutures are incorporated in it (Figure 5). Start the third layer of suturing from the middle and go in either direction. After taking two bites in each direction, pull-up sutures are knotted with the third layer suture on both sides.

10. Hepaticojejunostomy/choledochojejunostomy is performed proximally to GJ on the Roux limb.

A nasogastric tube was in place till POD 3. Patients were allowed to take oral liquids from POD 1 and a solid diet from POD 3. The nasojejunal tube was inserted in all patients for early enteral feeding and was operational from POD 1 till POD 5 and usually removed on POD 6. Out of eight patients, only one patient had Grade A POPF and none of the patients developed Grade B/C POPF. PPH was present in none of the patients. No delayed gastric emptying (DGE) was established in any of the patients. All the patients were discharged on or before 10 days and the readmission rate is zero. The 90-day mortality rate is zero.

## Discussion

PG has many theoretical advantages over PJ. Due to the proximity of the pancreas and stomach, anastomosis is easier to perform. The stomach has an excellent blood supply so theoretically, it has less chance of leaking. Due to the stomach's acidic environment, pancreatic juice is not activated. It is easier to learn and usually takes less or equal time than PJ and if any minor leak occurs it can be well managed by endoscopic interventions.

There are many risk factors associated with the development of POPF. The fistula risk score (FRS) of all patients is mentioned in the above table. High body mass index, soft texture of pancreas, small pancreatic duct diameter of ≤ 3 mm, drain amylase level at POD 3, excessive blood loss, and pancreatic pathology are some of the well-established risk factors [5,6].

Since the dawn of PG by Waugh and Clagett in 1946 [7], more than 20 modifications have been proposed by different authors, which consisted mainly of duct to mucosa sutures, use of stents, transfixing mattress sutures, purse string sutures, etc. [8-10]. Most of the authors have described their modified PG by creating gastrotomy both on the posterior and anterior walls of the stomach by using hemstitch sutures around the posterior gastrotomy or using two layers of purse string sutures. Few authors have used internal or external stents. Others have also fixed the pancreatic stump with the anterior seromuscular layer of the stomach [11-13].

In our technique, there is a two-layer safety of PG anastomosis. First, the purse string suture is tightened around PG, which ensures no leakage of stomach content out of the gastrotomy in the lesser sac. Second, trans-pancreatic sutures are secured with the third layer of GJ, which provides certainty that the pancreas will not get retracted back through posterior gastrotomy. Further, unlike most of the other techniques where there is a need to create a gastrotomy on the anterior layer of the stomach, we pass trans-pancreatic sutures through the already cut distal end of the stomach where future GJ anastomosis is done.

The above-described technique is easy to perform and learn. It is a handy technique, especially for teaching hospitals. A single abdominal drain like Jackson Pratt can be used in this technique since it can be passed underneath all three anastomoses of PD. The randomized controlled trials have not shown any difference between PG and PJ [14-17]. A meta-analysis done in 2019, which analyzed all randomized controlled trials of PG or PJ concluded that there is no difference in grade B/C POPF between the two techniques; however, PPH is higher in PG [18].

We usually remove the drain by POD 5 or 6, if the POD 3 drain amylase value is not more than three times the value of serum amylase on POD 3. Further, the content of drain output needs to be serous in nature. 

Several studies have shown that a non-dilated pancreatic duct or soft pancreas is a risk factor for leakage of PJ anastomosis [19,20]. In our series, there is no PPH, clinically significant POPF, or DGE. There is no readmission rate and 90 days mortality is zero although all the pancreas were soft and with non-dilated pancreatic ducts. This indicates that PG is suitable for the above risk factors and our two-layer safety technique is performing well.

There are limiting factors in our study like small sample size and retrospective analysis. Hence, a prospective study with a large sample size using this technique is needed to validate our findings.

## Conclusions

The described modification of PG with two transfixing sutures and one purse string suture and the incorporation of transfixing sutures in the third layer of the GJ is simple, safe, and sound. The presented retrospective series is small and, in the future, prospective studies and randomized controlled trials are needed to establish this technique as a practice-changing concept and to draw a clinically relevant conclusion.
